# Factors associated with successful transition from continuous renal replacement therapy in critically ill patients: a retrospective cohort study

**DOI:** 10.1080/0886022X.2025.2539933

**Published:** 2025-08-07

**Authors:** Ronald Miller, Abdel-Rauof Akkari, Ahmad Matarneh, Fareeha Khalil, Paddy Ssentongo, Sundus Sardar, Rafael Portela Colon, Mohammad Gul Khan, Yousif Rajab, Maaz Ali, Nadia Obaed, Samyukta Karthik, Medha Prabhu, Julio Hernandez, Yash Patel, Mujahed Dauleh, Grace Hwang, Gianni Pescetto, Nasrollah Ghahramani

**Affiliations:** a Division of Nephrology, Department of Medicine, Penn State Health, Milton S. Hershey Medical Center, Hershey, PA, USA; b Division of Infectious Diseases, Penn State Health, Milton S. Hershey Medical Center, Hershey, PA, USA; c Department of Internal Medicine, Penn State Health, Milton S. Hershey Medical Center, Hershey, PA, USA; d Penn State College of Medicine, Hershey, PA, USA; e Center for Kidney Disease and Hypertension, Wormleysburg, PA, USA

**Keywords:** Continuous renal replacement therapy, intermittent hemodialysis, acute kidney injury, dialysis transition, renal recovery, critical care nephrology

## Abstract

**Background:**

Acute kidney injury (AKI) in critically ill patients often requires continuous renal replacement therapy (CRRT), yet predictors of successful transition off CRRT remain unclear. This study aimed to identify clinical factors associated with successful and sustained CRRT discontinuation.

**Methods:**

We retrospectively analyzed 924 adult ICU patients who received CRRT between January 2010 and December 2024. The primary outcome was successful CRRT transition, defined as discontinuation without CRRT re-initiation within 7 days or transition to intermittent hemodialysis. Multivariable logistic regression was used to evaluate associations with clinical, hemodynamic, and biochemical parameters.

**Results:**

Of 924 patients (mean age 60 ± 14 years; 66% male), 823 (89%) successfully transitioned off CRRT. Vasopressor use (adjusted odds ratio [aOR] 0.68, *p* = 0.0001) and mechanical ventilation (aOR 0.56, *p* = 0.02) were associated with lower odds of success. On transition day, higher urine output (per 1 L, aOR 1.39, *p* = 0.003), serum bicarbonate (aOR 1.10, *p* = 0.032), and mean arterial pressure (aOR 1.02, *p* = 0.031) were positive predictors, while elevated blood urea nitrogen (aOR 0.98, *p* = 0.05) and higher obligate fluid intake (per 1 L, aOR 0.84, *p* = 0.032) were negative predictors. At day 7, sustained success was positively associated with urine output (aOR 1.35, *p* = 0.008) and serum pH (aOR 1.58, *p* = 0.049), and negatively associated with vasopressor use (aOR 0.69, *p* = 0.0001) and obligate intake (aOR 0.68, *p* < 0.0001).

**Conclusion:**

Higher urine output, better acid-base status, and stable hemodynamics predict successful and sustained CRRT discontinuation. Ongoing vasopressor use and high fluid burden reduce the likelihood of success.

## Introduction

Acute kidney injury (AKI) is a serious and often life-threatening condition, affecting approximately 30–60% of critically ill patients admitted to intensive care units (ICUs) [1]. AKI is defined as a sudden decline in renal function, resulting in the inability of the kidneys to effectively filter waste, regulate electrolytes, and maintain fluid balance [2]. This dysfunction can lead to severe complications, including fluid overload, metabolic disturbances, and multi-organ failure. Despite advancements in critical care, AKI remains a significant clinical challenge, contributing to prolonged hospital stays, increased morbidity, and high mortality rates [3]. While some patients recover renal function with supportive care alone, many require renal replacement therapy (RRT) to sustain life when endogenous kidney function becomes insufficient [4].

Among the primary modalities of RRT, intermittent hemodialysis (IHD) and continuous renal replacement therapy (CRRT) serve distinct patient populations [5]. IHD, performed over several hours per session, effectively clears toxins and corrects metabolic imbalances but requires hemodynamic stability, limiting its use in critically ill patients. Conversely, CRRT provides continuous solute and fluid removal, more closely mimicking native kidney function [6]. This approach is particularly beneficial for hemodynamically unstable patients, such as those with septic shock, cardiogenic shock, or severe volume overload, where abrupt fluid shifts could be detrimental.

The clinical outcomes of patients with AKI requiring CRRT vary widely. While some individuals successfully regain kidney function and discontinue dialysis, others develop chronic dialysis dependence, significantly affecting their quality of life and long-term survival [7]. Mortality rates among critically ill patients requiring CRRT remain high, exceeding 50%, particularly in those with sepsis, multi-organ dysfunction, or prolonged ICU stays [8]. Even among survivors, the risk of progression to chronic kidney disease (CKD) or end stage renal disease (ESRD) is considerable, necessitating long-term nephrology care.

The process of transitioning from CRRT to independent kidney function is multifaceted and influenced by several factors, including the severity and duration of AKI, baseline renal health, etiology of kidney injury, hemodynamic stability, and fluid balance. Patients with shorter CRRT durations, lower vasopressor requirements, and higher urine output exhibit a greater likelihood of renal recovery [9]. However, the precise predictors of successful CRRT discontinuation remain unclear, necessitating further investigation.

There is a paucity of evidence regarding the predictors of successful CRRT discontinuation, leading to a lack of standardized guidelines. This gap contributes to significant variability among physicians in determining the optimal timing for CRRT cessation. Addressing this issue is crucial to improving clinical decision-making and ensuring better patient outcomes.

This study aims to identify the key factors associated with successful transition from CRRT in patients with AKI. Understanding these predictors can enhance decision-making in the ICU, enabling clinicians to better assess which patients are likely to regain kidney function and discontinue dialysis. By analyzing clinical, biochemical, and hemodynamic data, this research seeks to refine risk stratification, optimize treatment strategies, and ultimately improve outcomes for patients undergoing CRRT. A clearer understanding of these predictive factors may facilitate earlier identification of patients suitable for CRRT discontinuation, thereby improving long-term recovery and quality of life in critically ill patients with AKI.

## Methods

### Study design and data collection

This retrospective observational study was conducted to identify clinical, biochemical, and hemodynamic factors associated with successful and sustained discontinuation of CRRT in critically ill patients with AKI. The aim was to evaluate parameters that can help predict (1) an initial successful transition from CRRT and (2) sustained success without requiring CRRT re-initiation within 7 days.

We utilized our institution’s electronic medical record (EMR) system to identify adult patients who received CRRT for AKI between 1 January 2010 and 31 December 2024. Patients were identified using Current Procedural Terminology (CPT) codes corresponding to continuous dialysis therapies. The study was approved by the institutional review board, and all data were de-identified prior to analysis.

### Patient selection and EMR review

The hospital’s EMR database was retrospectively searched using CPT codes for continuous dialysis to identify patients who received CRRT for AKI during the study period. The following CPT codes were utilized:

**Table:** 

CPT	Description of procedure
90945	Dialysis procedure other than hemodialysis
90947	Dialysis procedures other than non-hemodialysis

### Inclusion and exclusion criteria

Patients were eligible for inclusion if they were 18 years or older and had received CRRT for AKI. Exclusion criteria included patients younger than 18, those with preexisting ESRD requiring dialysis, and patients who were transferred out of the hospital while still on CRRT or within 7 days post-discontinuation. Patients were also excluded if they experienced a change in the level of care inconsistent with continued CRRT, such as transitioning to comfort care or withdrawal of life-sustaining therapy. as these changes reflect non-clinical decisions rather than clinical indicators of renal recovery. Other exclusions included death while on CRRT, being listed for a liver transplant, having previously received a kidney transplant, or undergoing CRRT for toxin removal rather than AKI management.

### Data collection, variables, and definition of successful transition from CRRT

For each patient, data were collected at multiple time points, including hospital admission, 48 h, and 24 h before CRRT discontinuation, on the day of discontinuation, and at 3 and 7 days post-discontinuation. At each time point, the following variables were collected: demographics, comorbidities (including diabetes and CKD), Sequential Organ Failure Assessment (SOFA) score, and hemodynamic parameters such as mean arterial pressure (MAP), vasopressor use which was defined by receiving norepinephrine or epinephrine or phenylephrine. The duration of pressors use in days was also included. Additionally, mechanical ventilation and mechanical circulatory support (e.g., extracorporeal membrane oxygenation and ventricular access devices). Also, metabolic and acid-base markers were recorded, including lactate, pH, bicarbonate, anion gap, serum creatinine, blood urea nitrogen (BUN) and potassium levels. Volume status was also assessed using fluid balance, body weight, and urine output. The study also documented diuretic use, which was defined as the administration of intravenous furosemide 80 mg, bumetanide 2 mg, or chlorthalidone 250 mg. A successful transition was defined as either complete discontinuation of CRRT without the need for further dialysis or a transition from CRRT to IHD. *Transition failure* was defined as the re-initiation of CRRT within 7 days following its initial discontinuation, regardless of whether the patient had been completely off dialysis or had transitioned to IHD during that period.

### Statistical analysis

Baseline characteristics were summarized using means and standard deviations for continuous variables and frequencies with percentages for categorical variables. Given the large sample size (*N* = 924), the distribution of continuous variables was assumed to approximate normality, and parametric methods were used for group comparisons. Differences between groups were assessed using two-tailed Student’s *t*-tests for continuous variables and chi-square or Fisher’s exact tests for categorical variables, as appropriate. The primary outcome was a successful transition from CRRT, defined as either complete discontinuation without subsequent dialysis or transition to IHD without re-initiation of CRRT within 7 days. A secondary outcome was sustained success, defined as remaining off CRRT through Day 7 post-discontinuation.

To identify independent predictors of successful CRRT discontinuation, multivariable logistic regression models were constructed at predefined clinical time points: hospital admission, 48 h prior, 24 h prior, and on the day of discontinuation (Day 0). Additional models evaluated sustained transition success at 3 and 7 days post-discontinuation. Candidate variables included demographics (age, sex), comorbidities (diabetes, CKD), severity of illness (SOFA score, Glasgow coma scale), organ support (mechanical ventilation, mechanical circulatory support, vasopressor duration and count), laboratory data (platelet count, bilirubin, serum lactate, pH, bicarbonate, anion gap, creatinine, BUN, potassium), and volume metrics (weight, urine output, and obligate fluid intake per liter). These variables were evaluated at each time point and considered for inclusion in the multivariable model. Variable selection for final models was conducted using bidirectional stepwise regression based on the Akaike information criterion. This method optimizes model fit while minimizing overfitting. All models were adjusted for age, sex, advanced CKD, SOFA score, and mechanical ventilation. Adjusted odds ratios (aORs) with 95% confidence intervals were reported for all associations. A two-sided *p*-value less than 0.05 was considered statistically significant. Statistical analyses were performed using R software (R Foundation for Statistical Computing, Vienna, Austria).

## Results

### Participant characteristics

A total of 924 patients met the inclusion criteria. Of these, 823 (89%) successfully transitioned off CRRT, while 101 (11%) experienced transition failure. The mean age was 60 ± 14 years, with no significant difference between the success and failure groups (60 ± 14.5 vs. 59 ± 14 years; *p* = 0.31). Two-thirds of the cohort were men, and the distribution of sex did not differ significantly between groups (66% vs. 74%; *p* = 0.17). The prevalence of advanced CKD (stage III or higher) was also similar (38% vs. 41%; *p* = 0.64). There was no significant difference in SOFA scores between the groups (6.6 ± 4.3 vs. 7.1 ± 4.3; *p* = 0.28). Mechanical ventilation was more common in the failure group than in the success group (71% vs. 57%; *p* = 0.01). Diabetes was more prevalent among patients who successfully transitioned off CRRT (41% vs. 28%; *p* = 0.02) (Table 1).

**Table 1.** Baseline characteristics of the study population stratified by CRRT transition outcome.

Variable	Overall *n* = 924	Successful transition *n* = 823	Unsuccessful transition *n* = 101	*p*-Value
Mean age, years ± SD	60 ± 14	60 ± 14.5	59 ± 14	0.31
Sex (male), *n* (%)	613 (66)	541 (66)	75 (74)	0.17
Diabetes mellitus, *n* (%)	365 (40)	336 (41)	29 (28)	0.02
Advanced CKD, *n* (%)	351 (38)	310 (38)	41 (41)	0.64
SOFA score (Mean ± SD)	6.7	6.6 ± 4.3	7.1 ± 4.3	0.28
Mechanical ventilation, *n* (%)	546 (59)	474 (57)	72 (71)	0.01

### Predictors of successful transition from CRRT

Multivariate logistic regression was performed to identify independent predictors of successful CRRT discontinuation. Results were analyzed by timepoint (Table 2).

**Table 2.** Factors associated with successful transition from CRRT, assessed prior to the day of discontinuation.

Variable	aOR	95% CI	*p*-value
Age	1.0120	0.9962–1.0281	0.1361
Mechanical ventilation	0.5586	0.3420–0.9124	0.02
Platelets count	0.9984	0.9966–1.0002	0.0826
Vasopressor	0.6828	0.5639–0.8268	0.0001
Lactate-admission	1.0817	0.9906–1.1812	0.0803
BUN-admission	0.9943	0.9876–1.0012	0.1038
Creatinine-admission	0.9979	0.9898–1.0061	0.6113
BUN (−48)	0.9862	0.9724–1.0001	0.0523
K (−48)	1.3212	0.8917–1.9578	0.1650
AG (−24)	1.0568	0.9909–1.1272	0.0926
BUN (−24)	1.0184	0.9948–1.0426	0.1281
K (−24)	0.9007	0.7421–1.0930	0.2894
Diuretics (−24)	0.6503	0.3878–1.0904	0.1027
MAP (D-0)	1.0209	1.0019–1.0402	0.0308
Bicarbonate (D-0)	1.0980	1.0047–1.1999	0.0392
BUN (D-0)	0.9816	0.9635–1	0.0495
UOP (D-0) per 1 L	1.3939	1.1172–1.7392	0.0033
Obligate intake (D-0) per 1 L	0.8429	0.7214–09850	0.0315

*Abbreviations:* −48 = 48 h before CRRT discontinuation; −24 = 24 h before discontinuation; D-0 = day of discontinuation; aOR = adjusted odds ratio; BUN = blood urea nitrogen; K = potassium; AG = anion gap; MAP = mean arterial pressure; UOP = urine output.

#### Forty-eight hours before CRRT discontinuation

At 48 h prior to CRRT discontinuation, we evaluated laboratory markers such as BUN, potassium, lactate, and others. BUN showed a borderline negative association with successful transition (OR 0.99, 95% CI 0.97–1.00, *p* = 0.0523), suggesting higher levels may portend lower likelihood of renal recovery. Lactate levels on admission were not statistically significant (OR 1.08, 95% CI 0.99–1.18, *p* = 0.0803), nor was serum potassium (OR 1.32, 95% CI 0.89–1.96, *p* = 0.165). Platelet count, admission creatinine, and other metabolic parameters were also not significantly associated with outcomes at this time point.

#### Twenty-four hours before CRRT discontinuation

At 24 h prior to discontinuation, the anion gap (OR 1.06, 95% CI 0.99–1.13, *p* = 0.0926), BUN (OR 1.02, *p* = 0.1281), and potassium (OR 0.90, *p* = 0.2894) continued to show no statistically significant associations with transition success. Diuretic administration at this timepoint—defined as a challenge dose of IV furosemide 80 mg, bumetanide 2 mg, or chlorthalidone 250 mg—was associated with lower odds of successful transition (OR 0.65, 95% CI 0.39–1.09, *p* = 0.10), though the finding was not statistically significant. These results suggest that diuretic use alone may not be a reliable marker of renal recovery.

#### On the day of CRRT discontinuation (D-0)

Several predictors measured on the day of CRRT discontinuation were significantly associated with successful transition. Higher urine output per liter was a strong positive predictor (OR 1.39, 95% CI 1.12–1.74, *p* = 0.0033), as was higher serum bicarbonate (OR 1.10, 95% CI 1.00–1.20, *p* = 0.0392) and MAP (OR 1.02, 95% CI 1.00–1.04, *p* = 0.031). These findings suggest that stable hemodynamics, adequate perfusion, and acid-base normalization are important markers of recovery.

In contrast, elevated BUN was negatively associated with successful discontinuation (OR 0.98, 95% CI 0.96–1.00, *p* = 0.0495), and high obligate fluid intake was also a negative predictor (OR 0.84 per liter, 95% CI 0.72–0.98, *p* = 0.0315), suggesting persistent azotemia and fluid overload may signal ongoing renal dysfunction. Diuretic use on the day of discontinuation was not directly assessed, but earlier diuretic response was not predictive.

### Predictors of sustained transition success

Next, we analyzed predictors of continued CRRT independence, defined as no re-initiation of CRRT within 7 days after initial discontinuation. Logistic regression models included variables from the day of transition and post-discontinuation Days 3 and 7 (Table 3).

**Table 3.** Factors associated with sustained successful transition from CRRT, assessed on the day of discontinuation and post-discontinuation Days 3 and 7.

Variable	aOR	95% CI	*p*-Value
Mechanical ventilation	0.6770	0.4173–1.0983	0.1141
Platelets count	0.9980	0.9962–0.9998	0.0280
Vasopressor	0.6920	0.5750–0.8329	0.0001
Mechanical circulatory support (D-0)	6.2307	0.5644–68.7862	0.1354
BUN (D-0)	0.9895	0.9789–1.0002	0.0547
UOP (D-0) per 1 L	1.2530	09999–1.5703	0.0501
K (D-3)	0.9540	0.8917–1.0203	0.1712
MAP (D-7)	1.0256	1.0073–1.0442	0.0059
Mechanical circulatory support (D-7)	0.1632	0.0207–1.2861	0.0852
PH (D-7)	1.5821	1.0015–2.4995	0.0493
UOP (D-7) per 1 L	1.3472	1.0805–1.6796	0.0081
Obligate intake (D-7) per 1 L	0.6824	0.5834–0.7970	0.0000

*Abbreviations:* D-0 = day of CRRT discontinuation; D-3 = third day post-discontinuation; D-7 = seventh day post-discontinuation; aOR = adjusted odds ratio; BUN = blood urea nitrogen; K = potassium; AG = anion gap; MAP = mean arterial pressure; UOP = urine output; pH = potential of hydrogen.

#### On the day of CRRT discontinuation (D-0)

Vasopressor use on the day of transition was a strong negative predictor of sustained success (OR 0.69, 95% CI 0.58–0.83, *p* = 0.0001), underscoring the importance of cardiovascular stability. Higher BUN on D-0 again showed a trend toward negative association (OR 0.99, 95% CI 0.98–1.00, *p* = 0.0547), though this did not reach statistical significance. Urine output remained a borderline significant positive predictor (OR 1.25, 95% CI 1.00–1.57, *p* = 0.0501). Platelet count was modestly but significantly associated with success (OR 0.998, 95% CI 0.996–0.9998, *p* = 0.028).

#### Post-discontinuation Day 3 (D-3)

No variables measured at this timepoint were significantly associated with sustained transition. Serum potassium levels, for example, were not predictive (OR 0.95, 95% CI 0.89–1.02, *p* = 0.1712). This suggests that Day 3 may not be an optimal time for early post-transition risk stratification.

#### Post-discontinuation Day 7 (D-7)

**Figure 1.**
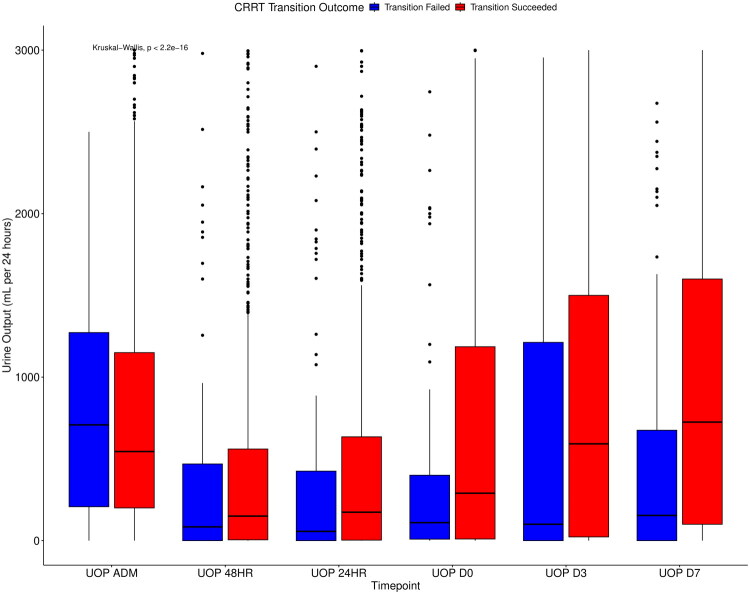
Urine output at key clinical timepoints stratified by CRRT transition outcome. Box plots display urine output (mL per 24 h) at six timepoints: admission (UOP ADM), 48 h before discontinuation (UOP 48HR), 24 h before discontinuation (UOP 24HR), on the day of discontinuation (UOP D0), and on post-discontinuation Days 3 and 7 (UOP D3 and UOP D7). Patients who successfully transitioned off CRRT are shown in red; those who failed transition (i.e., required CRRT re-initiation within 7 days) are shown in blue. *p*-Values reflect between-group comparisons at each timepoint. Median values, interquartile ranges, and outliers are visualized. Urine output remained consistently higher across all timepoints among those who successfully transitioned.

Day 7 measurements revealed multiple important predictors. Higher urine output remained a significant indicator of sustained success (OR 1.35 per liter increase, 95% CI 1.08–1.68, *p* = 0.0081), reinforcing its utility as a dynamic recovery marker (Figure 1). Similarly, higher serum pH was associated with success (OR 1.58, 95% CI 1.00–2.50, *p* = 0.0493), suggesting improved acid–base homeostasis correlates with renal stability. Elevated MAP also remained predictive (OR 1.03, 95% CI 1.01–1.04, *p* = 0.0059). In contrast, high obligate fluid intake on Day 7 significantly reduced the odds of sustained transition (OR 0.68 per liter increase, 95% CI 0.58–0.80, *p* < 0.0001). Mechanical circulatory support on Day 7 showed a non-significant trend toward poor outcome (OR 0.16, 95% CI 0.02–1.29, *p* = 0.0852).

## Discussion

AKI remains a significant challenge in critically ill patients, often necessitating CRRT. Despite its widespread use, standardized guidelines for CRRT discontinuation are lacking, leading to variability in clinical practice [10]. Our study identifies key clinical, biochemical, and hemodynamic parameters associated with successful transition from CRRT, offering potential insights to guide decision-making. Urine output regardless of diuretics use emerged as a pivotal factor in predicting a successful transition from CRRT. Patients who successfully transitioned off CRRT consistently demonstrated higher urine output at all measured time points, particularly on the day of transition and in the post-discontinuation period (Days 3 and 7). The statistically significant differences in urine output between the successful and unsuccessful groups emphasize its role as a strong predictor of renal recovery. Additionally, urine output per liter on the day of transition was independently associated with higher odds of successful transition (aOR 1.3939, *p* = 0.0033). These findings reinforce the importance of serial monitoring of urine output as a reliable marker of renal function improvement.

Hemodynamic stability also played a crucial role in CRRT discontinuation. A higher MAP on the day of transition and Day 7 post-transition was significantly associated with successful transition, underscoring the need for adequate perfusion for renal recovery. Use of vasopressors and mechanical ventilation remained independently associated with lower odds of successful CRRT transition, even after adjusting for SOFA score. Metabolic parameters further influenced the likelihood of successful discontinuation. A higher bicarbonate level on the day of transition was associated with improved transition outcomes suggesting that a normalized acid-base balance may signal improved systemic stability, including adequate perfusion and effective clearance. Conversely, elevated BUN levels on the day of transition were linked to lower odds of success indicating that persistent azotemia may reflect inadequate renal function for independent clearance.

Post-transition factors also played a role in sustained CRRT discontinuation success. Improved metabolic and hemodynamic parameters, including a higher pH and sustained urine output correlated with better renal recovery outcomes. These findings suggest that close monitoring beyond the initial transition period is essential for optimizing patient outcomes. These findings offer valuable insight into the physiologic and clinical parameters associated with successful CRRT discontinuation. To place our results in context, it is important to examine how they align with or differ from previously published studies. While there is growing interest in identifying objective markers for renal recovery, the existing literature remains limited—particularly regarding factors that predict sustained success after CRRT cessation.

Our results are largely consistent with prior studies evaluating predictors of successful CRRT discontinuation in critically ill patients with AKI. Several investigations have highlighted urine output as a key indicator of renal recovery. For example, Zhao et al. [1] and Zhang et al. [2] demonstrated that higher urine output at the time of CRRT discontinuation and during the following days correlated with greater likelihood of successful weaning. This closely mirrors our findings, in which urine output on the day of transition and on Day 7 post-discontinuation was strongly associated with successful outcomes. Similarly, vasopressor use has been shown to negatively impact transition outcomes. Studies by Jeon et al. [3] and Katayama et al. [4] reported lower success rates in patients requiring vasopressors, which aligns with our observation that vasopressor dependence significantly reduced the odds of sustained CRRT discontinuation.

In contrast to some prior research—such as the study by Srisawat et al. [5], which explored the predictive value of the furosemide stress test—our study did not find a significant association between diuretic use and successful transition. This may suggest that spontaneous urine output, rather than diuretic-induced diuresis, offers a more reliable reflection of true renal recovery in this patient population. Notably, our study adds to the existing literature by evaluating predictors beyond the point of CRRT cessation. We found that sustained urine output, higher serum pH, and improved MAP at Day 7 were associated with continued transition success. These findings underscore the importance of monitoring patients closely during the post-discontinuation period—a phase that has been underexplored in earlier studies. Finally, while most previous research has focused on patient-level variables, our study also highlights the potential impact of provider- and institution-level practice variation. Differences in clinical judgment regarding when to initiate or stop CRRT may affect outcomes and represent an important area for future standardization efforts.

## Clinical implications

Our findings highlight the need for a structured, parameter-driven approach to CRRT discontinuation. While no single variable can dictate the optimal timing for cessation, a combination of urine output trends, hemodynamic stability, and metabolic markers may improve clinical decision-making. By integrating these parameters, clinicians may be able to reduce unnecessary continuation of CRRT, optimize ICU resource utilization, and improve patient outcomes.

Furthermore, our study underscores the importance of careful patient selection when transitioning from CRRT to IHD, as some patients may require re-initiation of CRRT. Future research should focus on refining cutoff values for urine output, creatinine clearance, and other renal recovery markers to further standardize CRRT discontinuation protocols.

The consistent association of higher urine output, stable MAP, and improved acid-base status with successful CRRT transition suggests these parameters can guide clinicians in weaning decisions. Rather than relying on fixed thresholds, we recommend using trends in these markers to assess readiness. Sustained spontaneous urine output, especially volumes exceeding 0.5–1.0 L/day, may reflect recovering tubular function. Similarly, hemodynamic stability without vasopressor support and normalization of serum pH and bicarbonate may indicate sufficient systemic recovery to tolerate discontinuation. Together, these findings support a structured, physiology-based approach to CRRT weaning that complements clinical judgment and may reduce premature re-initiation or prolonged unnecessary therapy. Collectively, our findings support key observations from the literature while also contributing novel insights—particularly in the post-transition phase—and reinforce the need for standardized, data-driven protocols to guide CRRT discontinuation.

## Limitations and future directions

This study has several limitations. As a retrospective observational study, it is subject to selection bias, and causality cannot be established. Additionally, variability in clinical decision-making across different providers may have influenced the timing of CRRT discontinuation. Although we attempted to adjust for confounders, the absence of a standardized discontinuation protocol at our institution may limit the generalizability of our findings. Another limitation is that this study was conducted at a single institution, which may affect its applicability to other healthcare settings with different patient populations, CRRT discontinuation protocols, or ICU management strategies. A multicenter study would provide a more representative sample and enhance external validity. Furthermore, the lack of a predefined protocol for CRRT discontinuation may have introduced variability in clinical decisions. Differences in physician preferences, institutional practices, and patient-specific factors could have influenced transition success rates. We also acknowledge that our study did not account for potential differences in CRRT prescription parameters, including dose, modality and ultrafiltration rates, all of which may impact renal recovery and the transition success. Additionally, the duration of CRRT prior to discontinuation varied among patients, potentially influencing outcomes. While urine output was a major predictor, we did not comprehensively assess total fluid balance, cumulative fluid overload, or dynamic fluid removal trends during CRRT, all of which could significantly affect renal recovery and transition success.

Another important factor is the variation in practice patterns among providers at our institution. Some clinicians may feel more comfortable continuing therapy longer, while others may be more inclined to trial discontinuation earlier based on clinical improvement. These individual preferences, along with broader institutional and inter-hospital differences in CRRT initiation, prescription, and weaning criteria, likely influence patient outcomes. This highlights the need for standardized, evidence-based protocols to support consistent and effective decision-making.

Another key limitation is the exclusion of patients transferred out of the hospital shortly after CRRT discontinuation, as their long-term renal function outcomes were not captured. Our analysis primarily focused on short-term outcomes and did not evaluate long-term renal recovery, dialysis dependence, or mortality beyond hospitalization. Future prospective studies with extended follow-up are needed to determine whether the identified predictors correlate with sustained renal recovery and dialysis independence.

Given the observational nature of our study, we accounted for potential confounding by adjusting our regression models for variables such as age, sex, advanced CKD, SOFA score, and mechanical ventilation. Vasopressor use and mechanical ventilation remained independently associated with lower odds of successful CRRT transition, even after these adjustments. Additionally, mediation and interaction analyses demonstrated that these associations were not simply proxies for overall illness severity. While residual confounding cannot be fully excluded, these results strengthen the interpretation that urine output, MAP, and pH are independently useful markers of renal recovery.

Despite these limitations, our findings provide valuable insights into factors associated with successful CRRT discontinuation. Future research should focus on refining predictive models and exploring novel biomarkers to enhance clinical decision-making in critically ill patients requiring renal support.

## Conclusion

The decision to discontinue CRRT remains complex and requires a comprehensive assessment of renal function, hemodynamic stability, and metabolic recovery. Our study highlights key predictors that may help guide clinical decision-making, particularly urine output trends, renal function markers, and hemodynamic status. Additionally, we propose a predictive model that may enhance the objectivity and consistency of CRRT discontinuation decisions. Future efforts should focus on external validation, real-time model adaptation, and integration into clinical workflows to improve patient care. While further prospective research and external validation are needed, adopting a structured approach based on objective parameters and predictive modeling may help improve CRRT discontinuation strategies, reduce unnecessary resource utilization, and enhance outcomes for critically ill patients with AKI.

## Data Availability

The data used in this study are available upon reasonable request. Researchers interested in obtaining the data may contact the corresponding author for further details on the access process.

## Generative AI

Generative AI tools, including ChatGPT (OpenAI, GPT-4, May 2024 version), were used to assist with language refinement and formatting of the abstract and manuscript text. These tools were not used for data analysis, interpretation of findings, or generation of original scientific content. All outputs were reviewed and verified by the authors to ensure accuracy and integrity.
